# The efficacy and safety of Chinese herbal medicine in the treatment of painful diabetic neuropathy: A systematic review and meta-analysis

**DOI:** 10.3389/fphar.2023.1072991

**Published:** 2023-03-06

**Authors:** Min Song, Baogeng Huai, Zhenpeng Shi, Wenyi Li, Yutan Xi, Zhenguo Liu, Jihang Zhang, Junyu Zhou, Yun Qiao, Deshan Liu

**Affiliations:** ^1^ First Clinical Medical College, Shandong University of Traditional Chinese Medicine, Jinan, China; ^2^ Laboratory of Basic Medical Sciences, Qilu Hospital of Shandong University, Jinan, China; ^3^ Department of Oncology, Henan Provincial University of Traditional Chinese Medicine, Zhengzhou, China; ^4^ Department of Traditional Chinese Medicine, Qilu Hospital, Cheeloo College of Medicine, Shandong University, Jinan, China

**Keywords:** painful diabetic neuropathy, Chinese herbal medicine, efficacy, safety, systematic review, meta-analysis

## Abstract

**Objective:** The objective of this systematic review and meta-analysis is to assess the effectiveness and security of Chinese herbal medicine (CHM) in the therapy of painful diabetic neuropathy (PDN).

**Methods:** We searched databases for randomized controlled trials (RCTs) of CHM in the treatment of PDN. Outcome indicators included nerve conduction velocity, clinical efficiency, pain score, TCM syndrome score, and adverse events. Stata 16.0 was used to carry out the Meta-analysis.

**Results:** A total of 21 RCTs with 1,737 participants were included. This meta-analysis found that using CHM as adjuvant treatment or as monotherapy for PDN can improve SCV of median nerve [mean difference (MD) = 3.56, 95% Confidence interval (CI) (2.19, 4.92) ], MCV of median nerve [ MD = 3.82, 95% CI (2.51, 5.12) ], SCV of common peroneal nerve [ MD = 4.16, 95% CI (1.62, 6.70) ], MCV of common peroneal nerve [ MD = 4.37, 95% CI (1.82, 6.93) ], SCV of gastrocnemius nerve [ MD = 4.95, 95% CI (3.52, 6.37) ], SCV of tibial nerve [ MD = 3.17, 95% CI (−2.64, 8.99) ], MCV of tibial nerve [MD = 6.30, 95%CI (5.00, 7.60)] and clinical effective rate [ odds ratio (OR) = 4.00, 95% CI (2.89, 5.52) ] and reduce pain score [standardized mean difference (SMD) = -2.23, 95% CI (-3.04, -1.41) ], TCM syndrome score [ MD = -4.70, 95% CI (-6.61, -2.80) ]. In addition, compared to the control group, adverse events of Chinese medicine intervention occurred less.

**Conclusion:** CHM as adjuvant therapy or single treatment has a good curative effect and is safe for patients with PDN, which is worthy of clinical promotion and use, however; higher quality clinical studies are still needed to prove.

**Systematic Review Registration:** https://www.crd.york.ac.uk/, identifier CRD42022327967

## 1 Introduction

Painful diabetic neuropathy (PDN) is a common complication of type 2 diabetes, and about 16%–26% of diabetic patients will progress to PDN (Jensen et al., 2006; Tesfaye et al., 2011). The disease mostly starts from the distal limb, and its symptoms are mostly symmetrically distributed, with burning, electric shock, or acupuncture-like pain (Kulkantrakorn and Lorsuwansiri, 2013). The patient’s quality of life is drastically decreased by the pain, which gets worse over time, particularly at night, and may even lead to serious sleep disorders, anxiety, or depression (Veves et al., 2008; Sun et al., 2020). Despite considerable advancements in our knowledge of the pathophysiology of this condition, PDN is not currently managed with a specific medication (Duran et al., 2022). Presently, the clinical treatment of PDN refers to the combination of antidepressants, anticonvulsants, or opioids that are based on controlling blood glucose. These treatments result in only one-third of patients relieving half of their pain, while these treatments are often accompanied by serious side effects (Schreiber et al., 2015; Nayak et al., 2021). Due to the complexity and risk of disease, the development of an alternative or complementary therapies is urgent.

Chinese herbal medicine which is an essential part of traditional Chinese medicine, has been utilized successfully in China as a supplement and alternative form of therapy for thousands of years. Due to its “comprehensive, multi-channel, multi-target” therapy qualities, CHM has garnered increasing attention in the management of disease and comorbidities (Qinghua et al., 2019; Yue et al., 2019). In fact CHM has certain pharmacological effects mainly because of the active compounds contained in it. It has been found that the natural active compounds in CHM have a higher biological activity and structural diversity than artificial monomers, making it easier for them to enter the body and exert their medicinal effects, and possessing higher biological activity (Glevitzky et al., 2019; Fernandez-Ochoa et al., 2022).

In past decades, the number of RCTs assessing the safety and effectiveness of CHM as a single or adjuvant therapy for PDN has significantly expanded. However, there is still no systematic evaluation and meta-analysis of this issue to date. The purpose of this study is to systematically evaluate the efficacy and safety of CHM in the treatment of PDN, so as to provide high-quality evidence-based basis and treatment strategies for CHN in the treatment of PDN.

## 2 Methods

We completed this study using the Preferred Reporting Items for Systematic Review and Meta-Analysis (PRISMA) guidelines (Page et al., 2021) (Supplementary Material). In addition, the review has been registered in PROSPERO (CRD42022327967).

### 2.1 Literature search strategies

PubMed, Embase, The Cochrane Library, Web of Science, CNKI, Wanfang, VIP, and CBM were the eight databases we scanned through. All databases are available from their inception to 21 April 2022. The main search terms are: “Chinese herbal medicine”, “painful diabetic neuropathy”, “Chinese medicine”, and “diabetic neuropathic pain”.

### 2.2 Literature selection

Two researchers (ZP-S and WY-L) independently imported all the retrieved literature into the software EndnoteX9.0 for management and screening. For controversial literature, two researchers negotiate with a third researcher (YT-X).

The inclusion criteria were:1) participants: PDN patients (regardless of race, gender, or age); 2) study format: RCTs; 3) Interventions: the intervention group received CHM treatment (whether CHM as a single therapy or adjuvant therapy) and the control group used western medicine (WM) or placebo; and 4) outcomes: the main outcome indicators include SCV, MCV, pain score, and clinical efficiency The Secondary outcome indicators include TCM syndrome score and adverse events. The exclusion criteria were:1) treatment using acupuncture, massage, or other Chinese medicine; 2) Intervention time is not appropriate; and 3) patients without clear diagnostic criteria or accompanied by other diseases.

Herein, the definition of clinical efficiency in each trial is not the same, and the clinical efficiency of the included trials is based on the following criteria. a: Effectiveness: peripheral nerve function or clinical symptoms improved. b: Ineffectiveness: peripheral nerve function or clinical symptoms were not significantly improved or not improved (Xiaoyu, 2002).

### 2.3 Data extraction

Data was separately extracted and cross-checked by the two researchers (ZG-L and JH-Z). The extracted data primarily include: 1) the fundamental information of the selected research; 2) key elements of bias risk assessment; 3) outcome data: if the data type were measurement data, the mean and standard deviation were extracted, if the data were count data, the number of events and the total number were extracted.

### 2.4 Literature quality evaluation

Using the Risk of Bias instrument developed by the Cochrane Collaboration (Higgins et al., 2011), two researchers (MS and JY-Z) independently assessed the caliber of RCTs and cross-checked their results. The following seven components made up the evaluation content: the creation of random sequences, the concealment of allocations, the blinding of individuals and researchers, the integrity of outcome data, report bias, and other biases.

### 2.5 Statistical analysis

The meta-analysis of the research data was carried out using Stata16.0 software. The effect analysis statistic for categorical data was OR, while the effect analysis statistic for continuous data was either MD or SMD. For each effect, the 95% CI was calculated. The ***χ*** ^***2***^ test (test level = 0.10) was utilized to examine the heterogeneity of the outcomes of these studies, and the I^2^ test was used to quantify the heterogeneity. If *p*≥0.10 and I^2^<50%, the fixed effect model was used for analysis. If *p*<0.10 and I^2^ ≥ 50%, it indicates that a huge heterogeneity appeared among the studies, and then subgroup analyses have been conducted so as to make out the origin of heterogeneity. The analysis was carried out using a random effect model when methodological heterogeneity and clinical heterogeneity are absent. *α* = 0.05 was used as the meta-analysis test level. Through sensitivity analysis, the stability and reliability of the analysis’s results are examined. Publication bias was evaluated using the funnel plot, Begg’s test, and Egger’s test.

## 3 Results

### 3.1 Literature retrieval results

**FIGURE 1:**
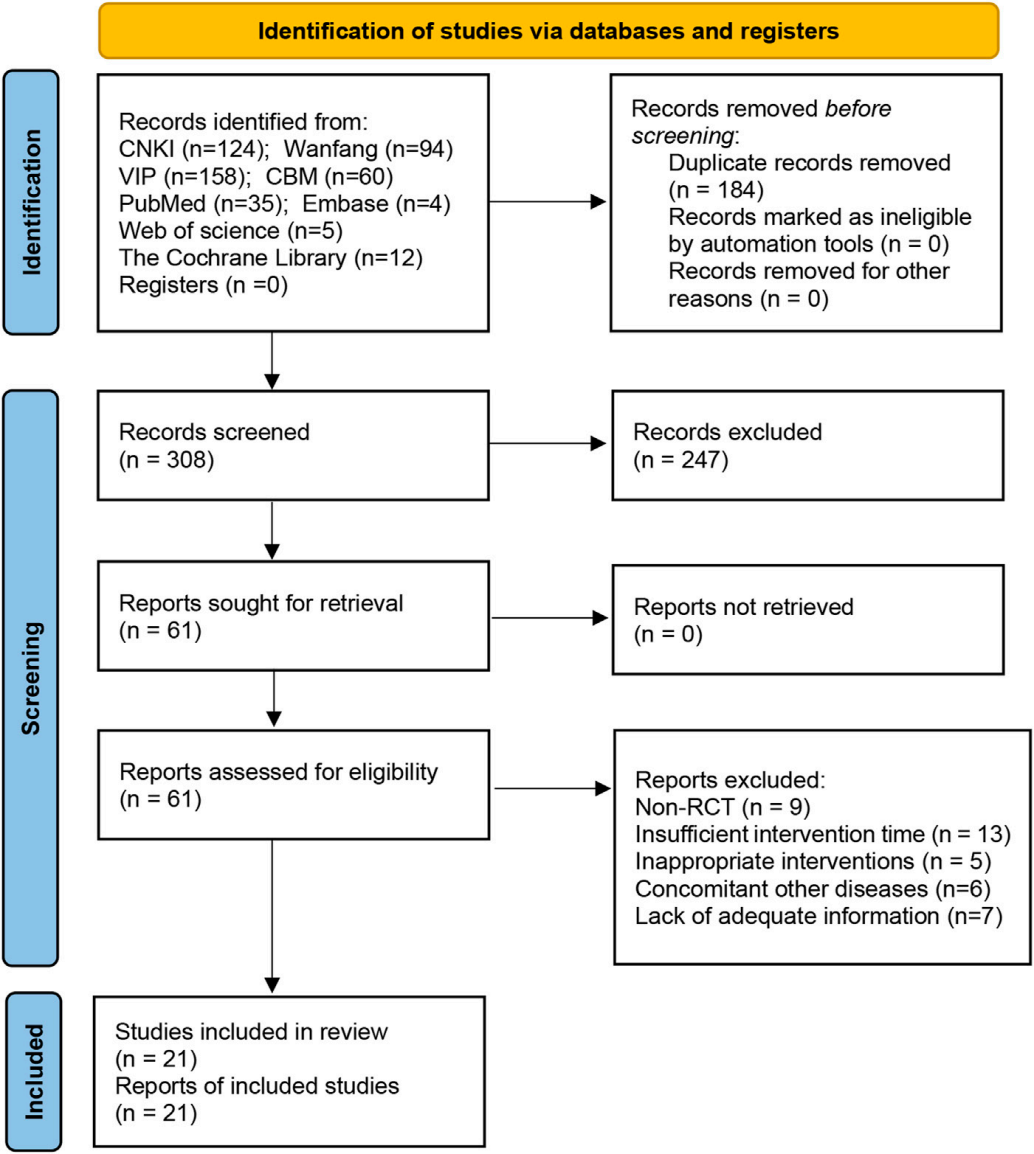
Prisma 2020 Flow Diagram.

A sum of 492 papers was found during the preliminary screening, however, 184 papers were eliminated due to repetition, 247 papers were eliminated by reading their titles and abstracts, and 40 papers were eliminated by reading the complete text. Finally, the quantitative analysis covered 21 articles (Hongwei et al., 2008; Honggang, 2011; Laibiao et al., 2012; Tsai et al., 2013; Fanrong and Mingkong, 2014; Laibiao et al., 2015; Ruixia et al., 2015; Haibo, 2016; Xiaorui, 2016; Di et al., 2017; Cizhen, 2018; Honggang et al., 2018; Liting et al., 2018; Qiaren and Xiaohong, 2018; Tao et al., 2018; Ye et al., 2018; Dianrong et al., 2020; Haiyan and Yange, 2020; Yanli et al., 2020; Qingqing, 2021; Shuquan et al., 2021). Figure 1 depicts the literature screening procedure.

### 3.2 Characteristics and quality evaluation of literature

There are 1737 patients altogether in the sample size of the 21 studies, which include 871 patients in the intervention group and 866 patients in the control group. The intervention group and the control group had equivalent pre-treatment data (such as age, sex ratio, outcome indicators, etc.). All 21 studies were from China (Table 1, Table 2).

**TABLE 1:** Basic information about the included literature.

Study	Study design	Diagnostic criteria	Sample size (male/female)		Mean age (year)		Interventions		Course of treatment	Outcome index
			Treatment group	Control group	Treatment group	Control group	Treatment group	Control group		
Cui XR 2016	RCT	B	35 (21, 14)	35 (23, 12)	57.69 ± 5.32	55.73 ± 6.28	Chaihu Shugan Powder	Duloxetine hydrochloride tablets	12 W	2, 3, 4, 5
Di HJ 2017	RCT	A	50 (24, 26)	50 (26, 24)	56.5 ± 7.1	55.5 ± 7.2	Shutangluo Compound + Epalrestat Tablets	Epalrestat Tablets	12 W	1, 2, 3
Ding LB 2012	RCT	B	30 (16, 14)	29 (15, 14)	55.3 ± 11.4	57.5 ± 10.8	Guilong Tongluo capsule	alprostadil injection	4 W	1, 2, 3
Ding LB 2015	RCT	B	40 (21, 19)	38 (20, 18)	55.3 ± 11.3	57.5 ± 10.6	Chuanwu decoction	alpha lipoic acid injection	4 W	1, 3, 5
Fang CZ 2018	RCT	B	44 (23, 21)	44 (24, 20)	52.6 ± 7.2	53.1 ± 7.3	Liuwei Dihuang decoction	carbamazepine	12 W	3
GU Y 2018	RCT	A	32 (18, 14)	30 (16, 14)	55.4 ± 6.6	52.2 ± 5.7	Buyang Huanwu decoction	Epalrestat Tablets	12 W	2, 3
He QR 2018	RCT	A	32 (17, 15)	32 (18, 14)	56.3 ± 4.6	56.1 ± 4.7	Shentong Zhuyu Decoction + Mecobalamin Tablets + Pregabalin CapsulPs	Mecobalamin Tablets + Pregabalin CapsulPs	6 W	1, 2, 3
Jia DR 2020	RCT	A	40 (23, 17)	40 (25, 15)	54.18 ± 3.54	55.02 ± 3.20	Tangbikang + Gabapentin	Gabapentin	8 W	1, 2, 3
Li FR 2014	RCT	B	100 (52, 48)	100 (58, 42)	54.14 ± 8.053	51.10 ± 9.399	classic prescription	Mecobalamin Injection	4 W	1, 2, 3
Li HW 2008	RCT	B	43 (22, 21)	43 (20, 23)	55.1 ± 11.7	54.4 ± 12.2	Self-made TCM prescription + Mecobalamin Tablets	Mecobalamin Tablets	4 W	1, 3
Liu HY 2020	RCT	A	46 (27, 19)	45 (25, 20)	60.10 ± 6.04	60.09 ± 6.02	Huoxue Tongbi decoction	Epalrestat Tablets	4 W	1, 2
Liu YL 2020	RCT	A	60 (33, 27)	60 (32, 28)	55.85 ± 7.14	56.25 ± 6.48	Mudan granules + Epalrestat Tablets + Mecobalamin Tablets	Epalrestat Tablets + Mecobalamin Tablets	4 W	2
Lv SQ 2021	RCT	A	30 ((16, 14)	30 (18, 12)	57.1 ± 5.5	57.3 ± 6.1	Wenyang Tongluo prescription + Gabapentin + EpalrestatCapsules	Gabapentin + EpalrestatCapsules	12 W	1, 3, 4, 5
Lv T 2018	RCT	A	30 (14, 16)	30 (15, 15)	46.8 ± 7.4	44.2 ± 7.1	Huangqi Guizhi Wuwu Decoction	basis treatment	8 W	1, 2, 3
Ma HB 2016	RCT	C	43 (22, 21)	43 (23, 20)	53.8 ± 1.9	54.1 ± 1.6	Yiqi Huoxue Tongmai Decoction	Phenytoin Sodium Tablets	4 W	1, 3
Ma QQ 2021	RCT	A	34 (22, 12)	36 (25, 11)	58.14士9.44	60.21士8.46	Jianpi Yishen Huayu Zhitong Prescription	Thioctic Acid Capsules	13 W	2, 4, 5
Ni HG 2011	RCT	B	24 (13, 11)	24 (13, 11)	56.1 ± 10.2	55.4 ± 9.7	Sigu Soup	Carbamazepine Tablets	4 W	2, 3
Ni HG 2018	RCT	C	27 (16, 11)	26 (16, 10)	56.1 ± 10.2	57.4 ± 10.3	Sigu Soup	Carbamazepine Tablets	4 W	1
Pei RX 2015	RCT	B	35 (20, 15)	35 (22, 13)	58.32 ± 4.68	57.24 ± 4.72	Chaihu Shugan Powder	Duloxetine Hydrochloride Enteric-coated Tablets	12 W	2, 4, 5
Tsai CI 2013	RCT	C	56 (29, 27)	56 (28, 28)	60.71 ± 10.20	60.46 ± 10.60	Huangqi Guizhi Wuwu Decoction	placebo	12 W	1, 2, 5
Zhao LT 2018	RCT	B	40 (23, 17)	40 (22, 18)	55.32 ± 2.21	55.39 ± 2.35	Sigu Soup + Carbamazepine Tablets	Carbamazepine Tablets	4 W	3

RCT: randomized controlled trial; A: China‘s guide; B: WHO guidelines; C: The US guide; 1: nerve conduction velocity (SCV + MCV); 2: pain score; 3: linical effective rates; 4: Chinese medicine syndrome scores; 5: adverse events.

**TABLE 2:** Compositions of Chinese Medicine Prescriptions in Each Study.

Study	Prescription name	Ingredients of herb prescription (Latin name)	Preparations
Cui XR 2016	Chaihu Shugan Powder	Bupleurum chinense DC. [Apiaceae; Bupleuri radix]12 g, Paeonia lactiflora Pall. [Paeoniaceae; Paeoniae radix rubra]15 g, Citrus × aurantium L. [Rutaceae; Aurantii fructus]12 g, *Cyperus* rotundus L. [Cyperaceae; Cyperi rhizoma]12 g, Curcuma aromatica Salisb. [Zingiberaceae; Curcumae radix]10 g, Cnidium monnieri (L.) Cusson [Apiaceae; Chuanxiong rhizoma]10 g, *Glycyrrhiza* inflata Batalin [Fabaceae; Glycyrrhizae radix et rhizoma praeparata cum melle]6 g, *Achyranthes* bidentata Blume [Amaranthaceae; Achyranthis bidentatae radix]15 g	Decoction
Di HJ 2017	Shutangluo Compound	Euonymus alatus (Thunb.)Sieb. [Celastraceae; Ramulus euonymi]10 g, Campsis grandiflora (Thunb.) K.Schum. [Bignoniaceae; Campsis flos]15 g, Rehmannia glutinosa (Gaertn.) DC. [Orobanchaceae; Rehmanniae radix]15 g, Coptis chinensis Franch. [Ranunculaceae; Coptidis rhizoma]3 g, Pueraria *montana* var. lobata (Willd.) Maesen & S.M.Almeida ex Sanjappa & Predeep [Fabaceae; Puerariae lobatae radix]15 g	Decoction
Ding LB 2012	Guilong Tongluo capsule	Neolitsea cassia (L.) Kosterm. [Lauraceae; Cinnamomi ramulus], Salvia miltiorrhiza Bunge [Lamiaceae; Salviae miltiorrhizae radix et rhizoma], Prunus persica (L.) Batsch [Rosaceae; Persicae semen], Carthamus tinctorius L. [Asteraceae; Carthami flos], Angelica sinensis (Oliv.) Diels [Apiaceae; Angelicae sinensis radix], Cnidium monnieri (L.) Cusson [Apiaceae; Chuanxiong rhizoma], Pheretima vulgaris Chen [Earthworms; Pheretima]	CHM capsules
Ding LB 2015	Chuanwu decoction	Aconitum carmichaelii Debeaux [Ranunculaceae; Aconiti radix cocta]30 g	Decoction
Fang CZ 2018	Liuwei Dihuang decoction	Cornus officinalis Siebold & Zucc. [Cornaceae; Corni fructus]12 g, Paeonia × suffruticosa Andrews [Paeoniaceae; Moutan cortex]10 g, Rehmannia glutinosa (Gaertn.) DC. [Orobanchaceae; Rehmanniae radix praeparata]15 g, *Dioscorea* oppositifolia L. [Dioscoreaceae; Dioscoreae rhizoma] 12 g, Wolfiporia cocos (F.A. Wolf) Ryvarden & Gilb. [Polyporus; Poria]10 g, Alisma plantago-aquatica L. [Alismataceae; Alismatis rhizoma]10 g	Decoction
GU Y 2018	Buyang Huanwu decoction	*Astragalus* mongholicus Bunge [Fabaceae; Astragali radix]30 g, Paeonia lactiflora Pall. [Paeoniaceae; Paeoniae radix rubra]15 g, Cnidium monnieri (L.) Cusson [Apiaceae; Chuanxiong rhizoma]15 g, Carthamus tinctorius L. [Asteraceae; Carthami flos]15 g, Prunus persica (L.) Batsch [Rosaceae; Persicae semen]10 g, Angelica sinensis (Oliv.) Diels [Apiaceae; Angelicae sinensis radix]15 g, Pheretima vulgaris Chen [Earthworms; Pheretima]15 g, Spatholobus suberectus Dunn [Fabaceae; Spatholobi caulis]30 g, Lycopodium japonicum Thunb. [Lycopodiaceae; Lycopodii herba]30 g, *Achyranthes* bidentata Blume [Amaranthaceae; Achyranthis bidentatae radix]15 g, Wolfiporia cocos (F.A. Wolf) Ryvarden & Gilb. [Polyporus; Poria]15 g, *Dioscorea* oppositifolia L. [Dioscoreaceae; Dioscoreae rhizoma] 15 g, *Glycyrrhiza* glabra L. [Fabaceae; Glycyrrhizae radix et rhizoma]10 g	Decoction
He QR 2018	Shentong Zhuyu Decoction	Prunus persica (L.) Batsch [Rosaceae; Persicae semen]15 g, Paeonia lactiflora Pall. [Paeoniaceae; Paeoniae radix rubra]15 g, Angelica sinensis (Oliv.) Diels [Apiaceae; Angelicae sinensis radix]15 g, Cnidium monnieri (L.) Cusson [Apiaceae; Chuanxiong rhizoma]15 g, Carthamus tinctorius L. [Asteraceae; Carthami flos]15 g, Commiphora myrrha (T.Nees) Engl. [Burseraceae; Myrrha]12 g, *Achyranthes* bidentata Blume [Amaranthaceae; Achyranthis bidentatae radix]12 g, Faeces Trogopterpri [Petauristidae; Faeces Trogopterpri]12 g, *Astragalus* mongholicus Bunge [Fabaceae; Astragali radix]30 g, *Cyperus* rotundus L. [Cyperaceae; Cyperi rhizoma]6 g, Hansenia forbesii (H.Boissieu) Pimenov & Kljuykov [Apiaceae; Notopterygii rhizoma et radix]6 g, *Gentiana* macrophylla Pall. [Gentianaceae; Gentianae macrophyllae radix]6 g, Pheretima vulgaris Chen [Earthworms; Pheretima]6 g, *Glycyrrhiza* glabra L. [Fabaceae; Glycyrrhizae radix et rhizoma]6 g	Decoction
Jia DR 2020	Tangbikang	*Astragalus* mongholicus Bunge [Fabaceae; Astragali radix]20 g, Angelica sinensis (Oliv.) Diels [Apiaceae; Angelicae sinensis radix]12 g, Cnidium monnieri (L.) Cusson [Apiaceae; Chuanxiong rhizoma]15 g, Panax notoginseng (Burkill) F.H.Chen [Araliaceae; Notoginseng radix et rhizoma]9 g, *Typha* angustifolia L. [Typhaceae; Typhae pollen]9 g, Corydalis yanhusuo (Y.H.Chou & Chun C.Hsu) W.T.Wang ex Z.Y.Su & C.Y.Wu [Papaveraceae; Corydalis rhizoma]12 g, Gypsophila vaccaria (L.) Sm. [Caryophyllaceae; Vaccariae semen]12 g, Scolopendra subspinipes mutilans L.Koch [Scolopendridae; Scolopendra]9 g, Pheretima vulgaris Chen [Earthworms; Pheretima]9 g, Neolitsea cassia (L.) Kosterm. [Lauraceae; Cinnamomi ramulus]12 g, Spatholobus suberectus Dunn [Fabaceae; Spatholobi caulis]12 g	Decoction
Li FR 2014	classic prescription	*Astragalus* mongholicus Bunge [Fabaceae; Astragali radix]50 g, Neolitsea cassia (L.) Kosterm. [Lauraceae; Cinnamomi ramulus]15 g, Paeonia lactiflora Pall. [Paeoniaceae; Paeoniae radix alba]50 g, Angelica sinensis (Oliv.) Diels [Apiaceae; Angelicae sinensis radix]10 g, Tetrapanax papyrifer (Hook.) K.Koch [Araliaceae; Tetrapanacis medulla]10 g, Asarum heterotropoides F.Schmidt [Aristolochiaceae; Asari radix et rhizoma]6 g, Cnidium monnieri (L.) Cusson [Apiaceae; Chuanxiong rhizoma]20 g, Alisma plantago-aquatica L. [Alismataceae; Alismatis rhizoma]20 g, Atractylodes macrocephala Koidz. [Asteraceae; Atractylodis macrocephalae rhizoma]20 g, Wolfiporia cocos (F.A. Wolf) Ryvarden & Gilb. [Polyporus; Poria]20 g, Bupleurum chinense DC. [Apiaceae; Bupleuri radix]10 g, Citrus × aurantium L. [Rutaceae; Aurantii fructus immaturus]20 g, Whitmania pigra Whitman [Hirudaceae; Hirudo]3 g, Prunus persica (L.) Batsch [Rosaceae; Persicae semen]10 g, Rheum palmatum L. [Polygonaceae; Rhei radix et rhizoma]5 g, *Glycyrrhiza* glabra L. [Fabaceae; Glycyrrhizae radix et rhizoma]5 g	Decoction
Li HW 2008	Self-made TCM prescription	Taxillus chinensis (DC.) Danser [Loranthaceae; Taxilli herba]30 g, Pseudostellaria heterophylla (Miq.) Pax [Caryophyllaceae; Pseudostellariae radix]30 g, Paeonia lactiflora Pall. [Paeoniaceae; Paeoniae radix alba]25 g, Cnidium monnieri (L.) Cusson [Apiaceae; Chuanxiong rhizoma]20 g, Wolfiporia cocos (F.A. Wolf) Ryvarden & Gilb. [Polyporus; Poria]20 g, *Dioscorea* collettii var. hypoglauca (Palib.) S.J.Pei & C.T.Ting [Dioscoreaceae; Dioscoreae spongiosae rhizoma]20 g, Coix lacryma-jobi L. [Poaceae; Coicis semen]20 g, Bupleurum chinense DC. [Apiaceae; Bupleuri radix]15 g, Cornus officinalis Siebold & Zucc. [Cornaceae; Corni fructus]15 g, *Achyranthes* bidentata Blume [Amaranthaceae; Achyranthis bidentatae radix]15 g, Ligustrum lucidum W.T.Aiton [Oleaceae; Ligustri lucidi fructus]15 g, Alisma plantago-aquatica L. [Alismataceae; Alismatis rhizoma]15 g, Panax notoginseng (Burkill) F.H.Chen [Araliaceae; Notoginseng radix et rhizoma]15 g, *Glycyrrhiza* glabra L. [Fabaceae; Glycyrrhizae radix et rhizoma]5 g	Decoction
Liu HY 2020	Huoxue Tongbi decoction	*Astragalus* mongholicus Bunge [Fabaceae; Astragali radix]30 g, Paeonia lactiflora Pall. [Paeoniaceae; Paeoniae radix alba]21 g, Atractylodes macrocephala Koidz. [Asteraceae; Atractylodis macrocephalae rhizoma]18 g, Cnidium monnieri (L.) Cusson [Apiaceae; Chuanxiong rhizoma]18 g, Citrus × aurantium L. [Rutaceae; Aurantii fructus immaturus]18 g, Wolfiporia cocos (F.A. Wolf) Ryvarden & Gilb. [Polyporus; Poria]18 g, Neolitsea cassia (L.) Kosterm. [Lauraceae; Cinnamomi ramulus]12 g, Bupleurum chinense DC. [Apiaceae; Bupleuri radix]12 g, Alisma plantago-aquatica L. [Alismataceae; Alismatis rhizoma]12 g, Tetrapanax papyrifer (Hook.) K.Koch [Araliaceae; Tetrapanacis medulla]9 g, Angelica sinensis (Oliv.) Diels [Apiaceae; Angelicae sinensis radix]9 g, Prunus persica (L.) Batsch [Rosaceae; Persicae semen]9 g, Rheum palmatum L. [Polygonaceae; Rhei radix et rhizoma]6 g, Asarum heterotropoides F.Schmidt [Aristolochiaceae; Asari radix et rhizoma]6 g, Whitmania pigra Whitman [Hirudaceae; Hirudo]6 g, *Glycyrrhiza* glabra L. [Fabaceae; Glycyrrhizae radix et rhizoma]6 g	Decoction
Liu YL 2020	Mudan granules	*Astragalus* mongholicus Bunge [Fabaceae; Astragali radix], Biancaea sappan (L.) Tod. [Fabaceae; Sappan lignum], Salvia miltiorrhiza Bunge [Lamiaceae; Salviae miltiorrhizae radix et rhizoma], Paeonia lactiflora Pall. [Paeoniaceae; Paeoniae radix rubra], Carthamus tinctorius L. [Asteraceae; Carthami flos], Panax notoginseng (Burkill) F.H.Chen [Araliaceae; Notoginseng radix et rhizoma], Spatholobus suberectus Dunn [Fabaceae; Spatholobi caulis], Corydalis yanhusuo (Y.H.Chou & Chun C.Hsu) W.T.Wang ex Z.Y.Su & C.Y.Wu [Papaveraceae; Corydalis rhizoma]	CHM granules
Lv SQ 2021	Wenyang Tongluo prescription	Ephedra sinica Stapf [Ephedraceae; Ephedrae herba]10 g, Aconitum carmichaelii Debeaux [Ranunculaceae; Aconiti lateralis radix praep arata]15 g, Asarum heterotropoides F.Schmidt [Aristolochiaceae; Asari radix et rhizoma]3 g, Neolitsea cassia (L.) Kosterm. [Lauraceae; Cinnamomi ramulus]12 g, *Glycyrrhiza* inflata Batalin [Fabaceae; Glycyrrhizae radix et rhizoma praeparata cum melle]10 g, Zingiber officinale Roscoe [Zingiberaceae; Zingiberis rhizoma recens]10 g, Buthus martensii Karsch [Buthidae; Scorpio]10 g, Scolopendra subspinipes mutilans L.Koch [Scolopendridae; Scolopendra]3 g, Paeonia lactiflora Pall. [Paeoniaceae; Paeoniae radix alba]30 g, *Astragalus* mongholicus Bunge [Fabaceae; Astragali radix]30 g, Angelica sinensis (Oliv.) Diels [Apiaceae; Angelicae sinensis radix]15 g, *Typha* angustifolia L. [Typhaceae; Typhae pollen]10 g, Faeces Trogopterpri [Petauristidae; Faeces Trogopterpri]10 g, Corydalis yanhusuo (Y.H.Chou & Chun C.Hsu) W.T.Wang ex Z.Y.Su & C.Y.Wu [Papaveraceae; Corydalis rhizoma]30 g, Rehmannia glutinosa (Gaertn.) DC. [Orobanchaceae; Rehmanniae radix]12 g	Decoction
Lv T 2018	Huangqi Guizhi Wuwu Decoction	*Astragalus* mongholicus Bunge [Fabaceae; Astragali radix]30 g, Neolitsea cassia (L.) Kosterm. [Lauraceae; Cinnamomi ramulus]15 g, Paeonia lactiflora Pall. [Paeoniaceae; Paeoniae radix alba]15 g, Paeonia lactiflora Pall. [Paeoniaceae; Paeoniae radix rubra]15 g, Salvia miltiorrhiza Bunge [Lamiaceae; Salviae miltiorrhizae radix et rhizoma]20 g, Spatholobus suberectus Dunn [Fabaceae; Spatholobi caulis]20 g, Buthus martensii Karsch [Buthidae; Scorpio]10 g, Corydalis yanhusuo (Y.H.Chou & Chun C.Hsu) W.T.Wang ex Z.Y.Su & C.Y.Wu [Papaveraceae; Corydalis rhizoma]15 g	Decoction
Ma HB 2016	Yiqi Huoxue Tongmai Decoction	Angelica sinensis (Oliv.) Diels [Apiaceae; Angelicae sinensis radix]15 g, Salvia miltiorrhiza Bunge [Lamiaceae; Salviae miltiorrhizae radix et rhizoma]15 g, Spatholobus suberectus Dunn [Fabaceae; Spatholobi caulis]15 g, Paeonia lactiflora Pall. [Paeoniaceae; Paeoniae radix alba]12 g, Cnidium monnieri (L.) Cusson [Apiaceae; Chuanxiong rhizoma]12 g, *Achyranthes* bidentata Blume [Amaranthaceae; Achyranthis bidentatae radix]12 g, Prunus persica (L.) Batsch [Rosaceae; Persicae semen]12 g, Carthamus tinctorius L. [Asteraceae; Carthami flos]12 g, Neolitsea cassia (L.) Kosterm. [Lauraceae; Cinnamomi ramulus]10 g, Massa medicata fermentata10 g, Gardenia jasminoides J.Ellis [Rubiaceae; Gardeniae fructus]6 g, *Glycyrrhiza* glabra L. [Fabaceae; Glycyrrhizae radix et rhizoma]6 g	Decoction
Ma QQ 2021	Jianpi Yishen Huayu Zhitong Prescription	*Astragalus* mongholicus Bunge [Fabaceae; Astragali radix]32 g, *Achyranthes* bidentata Blume [Amaranthaceae; Achyranthis bidentatae radix]15 g, Rehmannia glutinosa (Gaertn.) DC. [Orobanchaceae; Rehmanniae radix]15 g, Rehmannia glutinosa (Gaertn.) DC. [Orobanchaceae; Rehmanniae radix praeparata]15 g, Codonopsis pilosula (Franch.) Nannf. [Campanulaceae; Codonopsis radix]20 g, Polygonatum sibiricum Redouté [Asparagaceae; Polygonati rhizoma]10 g, Lycium barbarum L. [Solanaceae; Lycii fructus]15 g, Neolitsea cassia (L.) Kosterm. [Lauraceae; Cinnamomi cortex]5 g, Alisma plantago-aquatica L. [Alismataceae; Alismatis rhizoma]10 g, Dendrobium nobile Lindl. [Orchidaceae; Dendrobii caulis]12 g, Angelica sinensis (Oliv.) Diels [Apiaceae; Angelicae sinensis radix]15 g, Wolfiporia cocos (F.A. Wolf) Ryvarden & Gilb. [Polyporus; Poria]12 g, *Bombyx mori* Linnaeus [Bombycidae; *Bombyx* batryticatus]10 g, Cnidium monnieri (L.) Cusson [Apiaceae; Chuanxiong rhizoma]15 g, Paeonia lactiflora Pall. [Paeoniaceae; Paeoniae radix rubra]15 g, Pheretima vulgaris Chen [Earthworms; Pheretima]15 g, Carthamus tinctorius L. [Asteraceae; Carthami flos]10 g, Prunus persica (L.) Batsch [Rosaceae; Persicae semen]15 g, Schisandra chinensis (Turcz.) Baill. [Schisandraceae; Schisandrae chinensis fructus]12 g, Ophiopogon japonicus (Thunb.) Ker Gawl. [Asparagaceae; Ophiopogonis radix]12 g	Decoction
Ni HG 2011	Sigu Soup	*Lonicera japonica* Thunb. [Caprifoliaceae; Lonicerae japonicae flos]45 g, *Astragalus* mongholicus Bunge [Fabaceae; Astragali radix]30 g, Scrophularia ningpoensis Hemsl. [Scrophulariaceae; Scrophulariae radix]30 g, Angelica sinensis (Oliv.) Diels [Apiaceae; Angelicae sinensis radix]12 g, *Achyranthes* bidentata Blume [Amaranthaceae; Achyranthis bidentatae radix]15 g, *Glycyrrhiza* glabra L. [Fabaceae; Glycyrrhizae radix et rhizoma]10 g, Adenophora triphylla (Thunb.) A.DC. [Campanulaceae; Adenophorae radix]30 g, Paeonia lactiflora Pall. [Paeoniaceae; Paeoniae radix rubra]30 g, Salvia miltiorrhiza Bunge [Lamiaceae; Salviae miltiorrhizae radix et rhizoma]30 g, Corydalis yanhusuo (Y.H.Chou & Chun C.Hsu) W.T.Wang ex Z.Y.Su & C.Y.Wu [Papaveraceae; Corydalis rhizoma]15 g, Curcuma longa L. [Zingiberaceae; Curcumae longae rhizoma]10 g, Spatholobus suberectus Dunn [Fabaceae; Spatholobi caulis]30 g	Decoction
Ni HG 2018	Sigu Soup	*Lonicera japonica* Thunb. [Caprifoliaceae; Lonicerae japonicae flos]45 g, *Astragalus* mongholicus Bunge [Fabaceae; Astragali radix]30 g, Scrophularia ningpoensis Hemsl. [Scrophulariaceae; Scrophulariae radix]30 g, Angelica sinensis (Oliv.) Diels [Apiaceae; Angelicae sinensis radix]12 g, *Achyranthes* bidentata Blume [Amaranthaceae; Achyranthis bidentatae radix]15 g, *Glycyrrhiza* glabra L. [Fabaceae; Glycyrrhizae radix et rhizoma]10 g, Adenophora triphylla (Thunb.) A.DC. [Campanulaceae; Adenophorae radix]30 g, Paeonia lactiflora Pall. [Paeoniaceae; Paeoniae radix rubra]30 g, Salvia miltiorrhiza Bunge [Lamiaceae; Salviae miltiorrhizae radix et rhizoma]30 g, Corydalis yanhusuo (Y.H.Chou & Chun C.Hsu) W.T.Wang ex Z.Y.Su & C.Y.Wu [Papaveraceae; Corydalis rhizoma]15 g, Curcuma longa L. [Zingiberaceae; Curcumae longae rhizoma]10 g, Spatholobus suberectus Dunn [Fabaceae; Spatholobi caulis]30 g	Decoction
Pei RX 2015	Chaihu Shugan Powder	Bupleurum chinense DC. [Apiaceae; Bupleuri radix], Citrus × aurantium L. [Rutaceae; Aurantii fructus], Paeonia lactiflora Pall. [Paeoniaceae; Paeoniae radix rubra], *Glycyrrhiza* glabra L. [Fabaceae; Glycyrrhizae radix et rhizoma], *Cyperus* rotundus L. [Cyperaceae; Cyperi rhizoma], Citrus × aurantium f. deliciosa (Ten.) M.Hiroe [Rutaceae; Citri reticulatae pericarpium], Pheretima vulgaris Chen [Earthworms; Pheretima], *Achyranthes* bidentata Blume [Amaranthaceae; Achyranthis bidentatae radix], Cnidium monnieri (L.) Cusson [Apiaceae; Chuanxiong rhizoma]	Decoction
Tsai CI 2013	Huangqi Guizhi Wuwu Decoction	*Astragalus* mongholicus Bunge [Fabaceae; Astragali radix], Neolitsea cassia (L.) Kosterm. [Lauraceae; Cinnamomi ramulus], Paeonia lactiflora Pall. [Paeoniaceae; Paeoniae radix alba], Zingiber officinale Roscoe [Zingiberaceae; Zingiberis rhizoma recens], Ziziphus jujuba Mill. [Rhamnaceae; Jujubae fructus], Pheretima vulgaris Chen [Earthworms; Pheretima], Spatholobus suberectus Dunn [Fabaceae; Spatholobi caulis]	Decoction
Zhao LT 2018	Sigu Soup	*Lonicera japonica* Thunb. [Caprifoliaceae; Lonicerae japonicae flos]45 g, *Astragalus* mongholicus Bunge [Fabaceae; Astragali radix]30 g, Scrophularia ningpoensis Hemsl. [Scrophulariaceae; Scrophulariae radix]30 g, Angelica sinensis (Oliv.) Diels [Apiaceae; Angelicae sinensis radix]12 g, *Achyranthes* bidentata Blume [Amaranthaceae; Achyranthis bidentatae radix]15 g, *Glycyrrhiza* glabra L. [Fabaceae; Glycyrrhizae radix et rhizoma]10 g, Adenophora triphylla (Thunb.) A.DC. [Campanulaceae; Adenophorae radix]30 g, Paeonia lactiflora Pall. [Paeoniaceae; Paeoniae radix rubra]30 g, Salvia miltiorrhiza Bunge [Lamiaceae; Salviae miltiorrhizae radix et rhizoma]30 g, Corydalis yanhusuo (Y.H.Chou & Chun C.Hsu) W.T.Wang ex Z.Y.Su & C.Y.Wu [Papaveraceae; Corydalis rhizoma]15 g, Curcuma longa L. [Zingiberaceae; Curcumae longae rhizoma]10 g, Spatholobus suberectus Dunn [Fabaceae; Spatholobi caulis]30 g	Decoction

**FIGURE 2:**
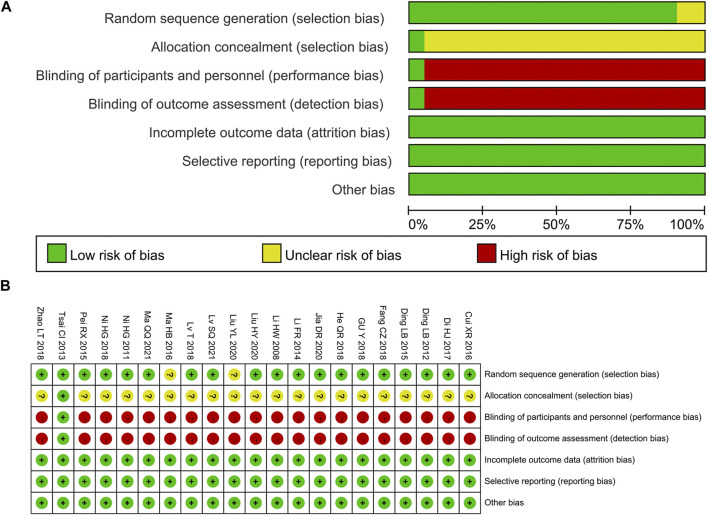
Risk-of-bias **(A)** Risk-of-bias graph; **(B)** Risk-of-bias summary.

Among the 21 studies included, 7 studies (Tsai et al., 2013; Fanrong and Mingkong, 2014; Ruixia et al., 2015; Di et al., 2017; Cizhen, 2018; Tao et al., 2018; Shuquan et al., 2021) were grouped by a random number table, 1 study (Yanli et al., 2020)used a draw for grouping, 1 study (Haibo, 2016) adopted the odd-even number method for grouping, and the remaining studies did not explain the specific methods. The allocation concealment method was not discussed in any of the 21 studies. One study (Tsai et al., 2013) used double-blind, and the remaining research failed to state whether or not blinding was utilized. All studies’ outcome data were complete, and there were no other biases or selective reporting results (Figure 2).

### 3.3 Results of meta-analysis

#### 3.3.1 Pain score

**FIGURE 3:**
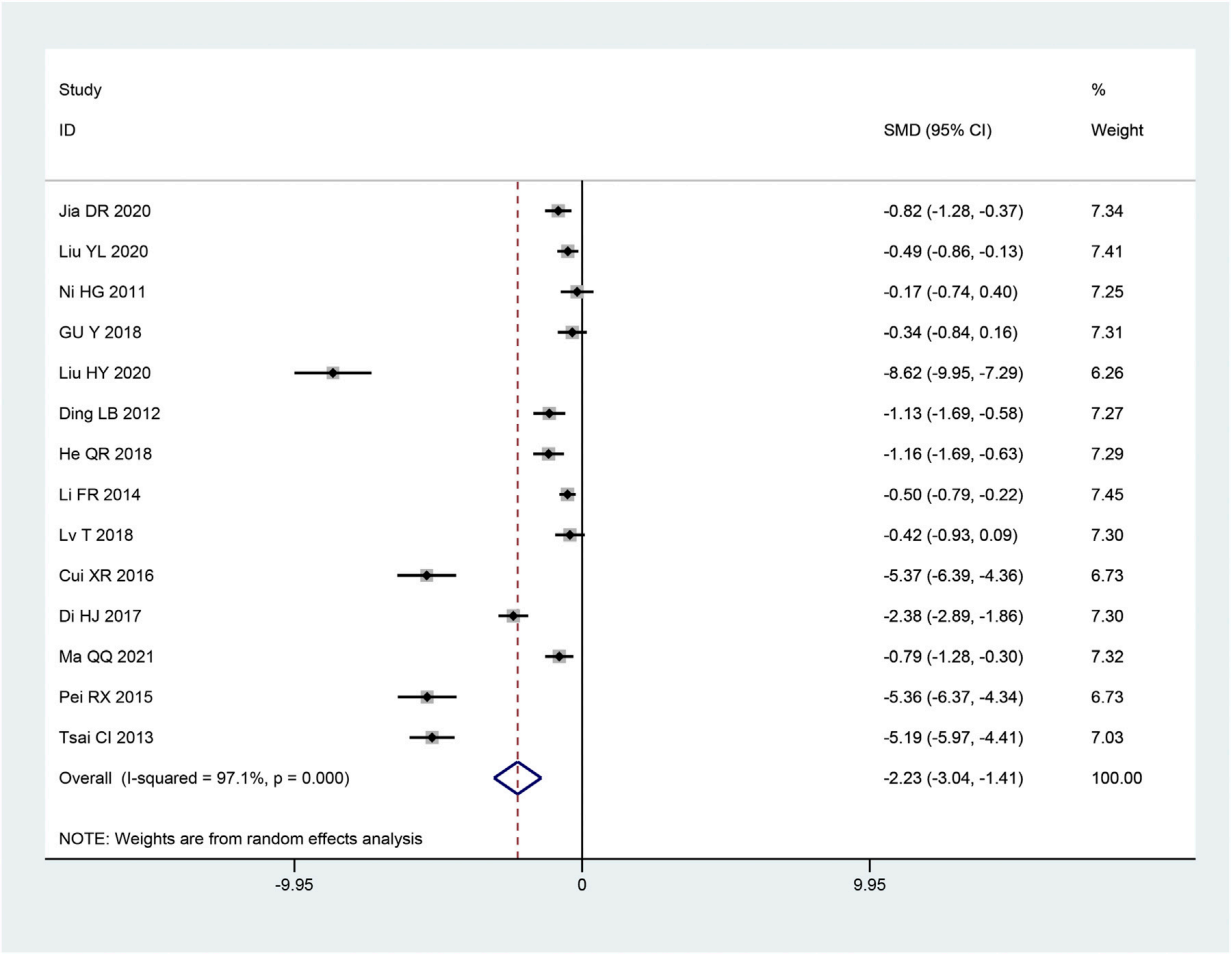
Forest plot of Pain Score.

14 articles (Honggang, 2011; Laibiao et al., 2012; Tsai et al., 2013; Fanrong and Mingkong, 2014; Ruixia et al., 2015; Xiaorui, 2016; Di et al., 2017; Qiaren and Xiaohong, 2018; Tao et al., 2018; Ye et al., 2018; Dianrong et al., 2020; Haiyan and Yange, 2020; Yanli et al., 2020; Qingqing, 2021) talked about pain scores. The studies’ significant heterogeneity was shown using the heterogeneity test (*p* < 0.10, I^2^ = 97.1%). Thus, to combine the effect sizes, we employ a random effects model. The outcomes demonstrated that, in comparison to the control group, the intervention group was able to significantly reduce pain and increase pain scores and the change was statistically meaningful [SMD = − 2.23, 95% CI (−3.04, − 1.41), *p* < 0.05; Figure 3]. The kind of pain scale and the length of the intervention were significantly different among subgroups according to subgroup analysis (*p* < 0.05 and *p* < 0.05, respectively). But the sample size and intervention strategies did not significantly differ (*p* = 0.566 and *p* = 0.48, respectively) (Table 3).

**TABLE 3:** Subgroup analysis for outcomes.

	Number of studies	*p-*value of subgroup difference	Heterogeneity test		Analysis results	p-value for overall effect
			I^2^ (%)	*p*-Value		
Intervention time		<0.05				
≥12 W	2		0	0.917	MD = 0.773 (-0.821, 2.367)	<0.01
<12 W	8		78.3	<0.1	MD = 4.148 (2.831, 5.466)	0.342
Intervention types		<0.05				
simple CHM	5		0	0.536	MD = 5.67 (4.96, 6.37)	<0.01
CHM + WM	5		9.5	0.352	MD = 2.04 (1.23, 2.85)	<0.01
sample size		0.003				
≥100	2		96.5	<0.1	MD = 3.52 (-1.84, 8.88)	0.198
<100	8		50.3	0.05	MD = 3.52 (2.47, 4.56)	<0.01
MCV of median nerve						
Intervention types		0.121				
simple CHM	5		85.3	<0.1	MD = 3.56 (1.87, 5.24)	<0.01
CHM + WM	3		58.1	0.092	MD = 3.82 (2.51, 5.12)	<0.01
sample size		<0.05				
≥100	1		—	—	MD = 0.90 (-0.12, 1.92)	0.084
<100	7		1.1	0.416	MD = 4.19 (3.54, 4.84)	<0.01
SCV of common peroneal nerve						
Intervention time		<0.05				
≥12 W	1		—	—	MD = 0.72 (-0.91, 2.35)	0.386
<12 W	8		95.7	<0.1	MD = 4.60 (1.93, 7.27)	0.001
Intervention types		<0.05				
simple CHM	5		97.1	<0.1	MD = 5.40 (1.50, 9.30)	0.007
CHM + WM	4		60.9	0.053	MD = 2.51 (1.04, 3.97)	0.001
sample size		<0.05				
≥100	2		80.3	0.024	MD = -0.57 (-3.03, 1.90)	0.653
<100	7		90.5	<0.1	MD = 5.57 (3.61, 7.54)	<0.01
MCV of common peroneal nerve						
Intervention time		0.056				
≥12 W	1		—	—	MD = -13.71 (-31.55, 5.13)	0.154
<12 W	8		95	<0.1	MD = 4.67 (2.12, 7.22)	<0.01
Intervention types		<0.05				
simple CHM	6		95.9	<0.1	MD = 4.99 (1.30, 8.67)	0.008
CHM + WM	3		58.5	<0.1	MD = 2.99 (1.16, 4.83)	0.001
sample size		<0.05				
≥100	2		56	0.132	MD = -3.28 (-16.07, 9.51)	0.615
<100	7		92.3	<0.1	MD = 5.26 (2.87, 7.66)	<0.01
pain score						
Type of scale		<0.05				
NRS	3		37	0.204	SMD = -0.53 (-0.79, -0.28)	<0.01
BPI-DPN	2		99.2	<0.1	SMD = -4.45 (-122.57, 3.66)	0.282
VAS	9		97.2	<0.1	SMD = -2.42 (-3.48, -1.35)	<0.01
Intervention time		<0.05				
≥12 W	6		97.7	<0.1	SMD = -3.20 (-4.93, -1.47)	<0.01
<12 W	8		95.3	<0.1	SMD = -1.44 (-2.22, -0.67)	<0.01
Intervention types		0.48				
simple CHM	10		91.6	<0.1	SMD = -2.70 (-3.90, -1.49)	<0.01
CHM + WM	4		97.8	<0.1	SMD = -1.20 (-1.99, -0.41)	0.003
sample size		0.566				
≥100	4		98.1	<0.1	SMD = -2.10 (-3.65, -0.55)	0.008
<100	10		96.9	<0.1	SMD = -2.30 (-3.37, -1.24)	<0.01
TCM syndrome scores						
Intervention types		0.009				
simple CHM	3		45.1	0.162	MD = -5.41 (-6.34, -4.49)	<0.01
CHM + WM	1		—	—	MD = -2.26 (-4.44, -0.08)	0.042

#### 3.3.2 Nerve conduction velocity

##### 3.3.2.1 SCV of median nerve

**FIGURE 4:**
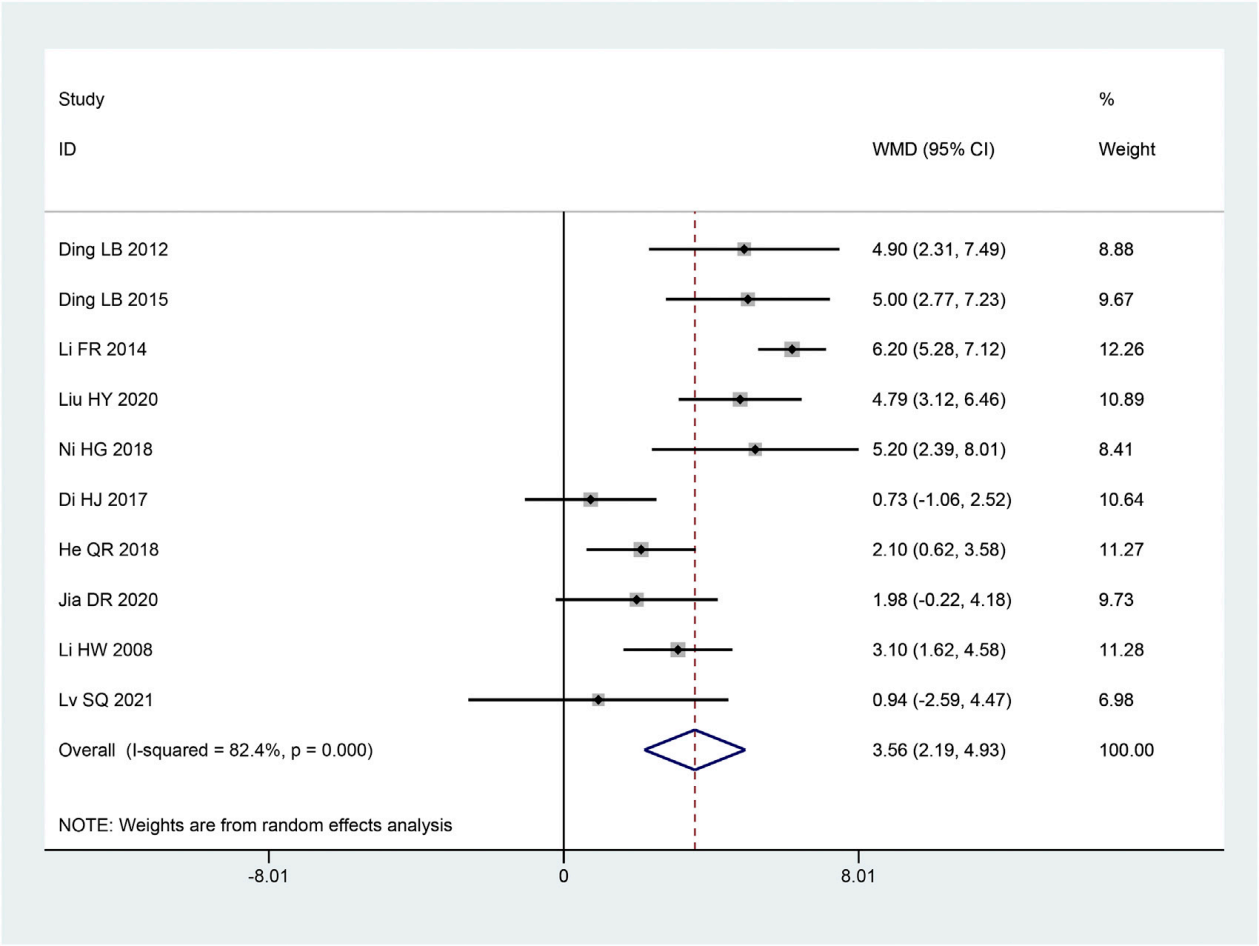
Forest plot of SCV of median nerve.

The SCV of median nerve was reported in 10 articles (Hongwei et al., 2008; Laibiao et al., 2012; Fanrong and Mingkong, 2014; Laibiao et al., 2015; Di et al., 2017; Honggang et al., 2018; Qiaren and Xiaohong, 2018; Dianrong et al., 2020; Haiyan and Yange, 2020; Shuquan et al., 2021). Following the heterogeneity test, there was significant homogeneity between the papers (*p* < 0.10, I^2^ = 82%). The results of a random effect model that combined effect sizes revealed that the intervention group considerably improved SCV of the median nerve compared to the control group, and the results of this experiment were statistically significant [MD = 3.56, 95% CI (2.19, 4.92), *p* < 0.05; Figure 4]. According to subgroup analysis, there were significant differences across subgroups with various intervention times (*p* < 0.05), sample sizes (*p* = 0.003), and intervention methods (*p* < 0.05) (Table 3).

##### 3.3.2.2 MCV of median nerve

**FIGURE 5:**
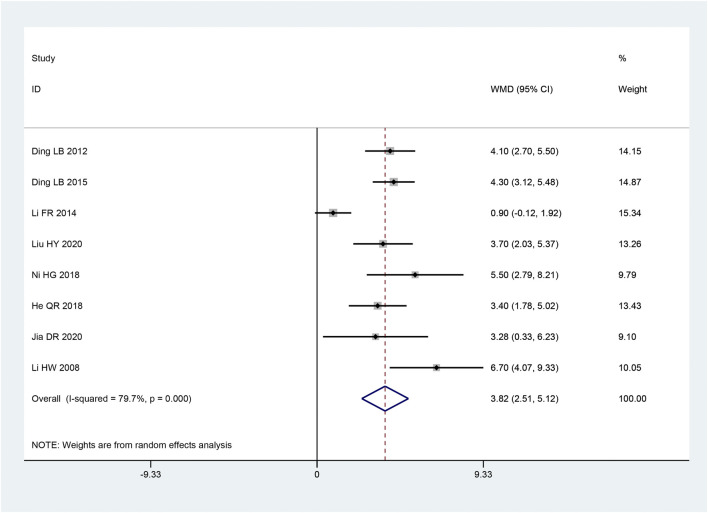
Forest plot of MCV of median nerve.

The MCV of median nerve appeared in eight articles (Hongwei et al., 2008; Laibiao et al., 2012; Fanrong and Mingkong, 2014; Laibiao et al., 2015; Honggang et al., 2018; Qiaren and Xiaohong, 2018; Dianrong et al., 2020; Haiyan and Yange, 2020). Based on the heterogeneity test, there was significant heterogeneity amongst the data (*p* < 0.10, I^2^ = 79.7%). The improvement of the MCV of the median nerve was discovered to be better in the intervention group than in the control group, and this experiment’s findings were statistically significant. [MD = 3.82, 95% CI (2.51, 5.12), *p* < 0.05; Figure 5] by the random effect model combining with the effect size. There were obvious differences between sample sizes (*p* < 0.05), as revealed by subgroup analysis, but there was no statistically significant difference between the intervention groups (*p* = 0.121) (Table 3).

##### 3.3.2.3 SCV of common peroneal nerve

**FIGURE 6:**
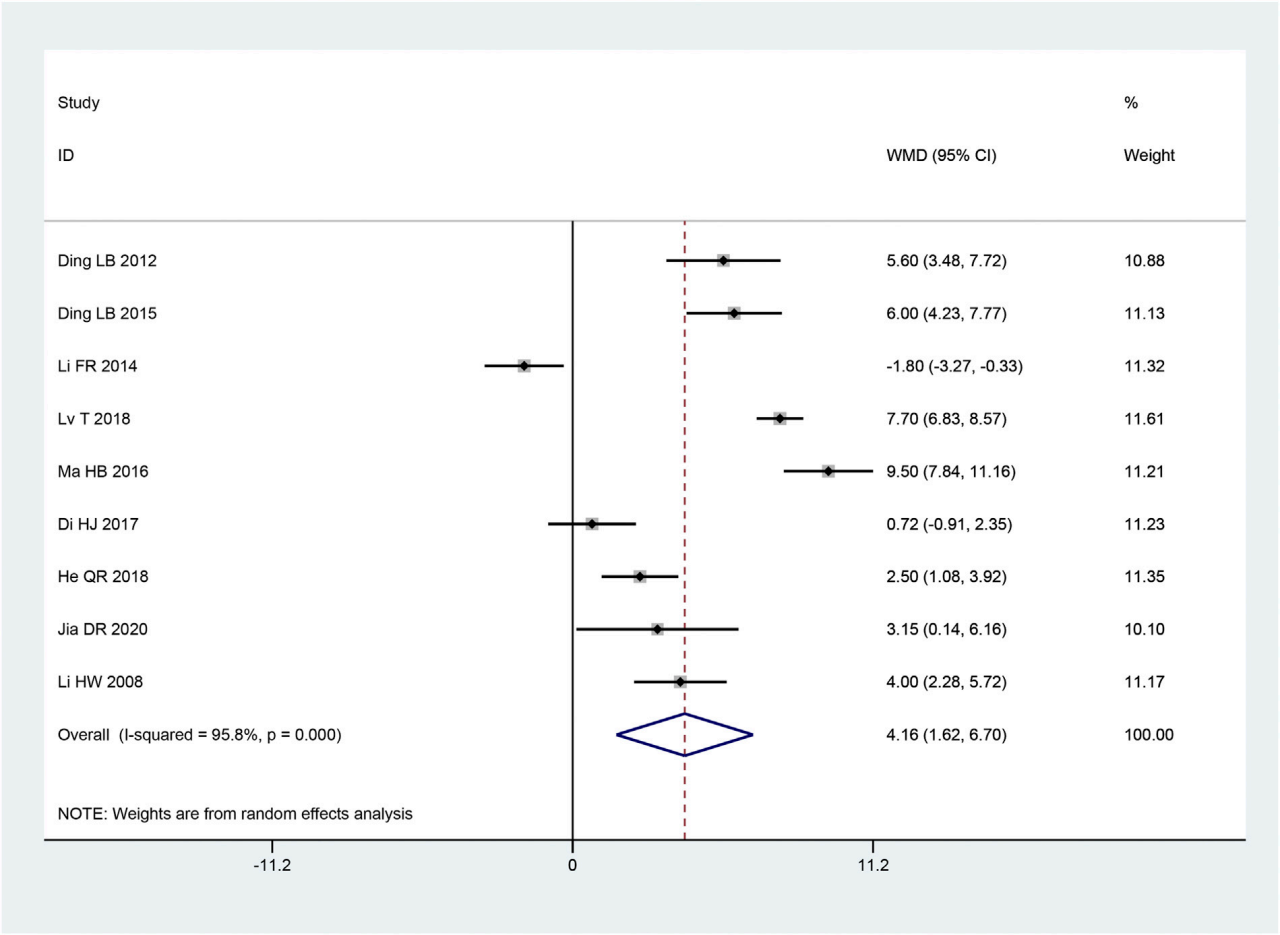
Forest plot of SCV of Common Peroneal Nerve.

Nine articles (Hongwei et al., 2008; Laibiao et al., 2012; Fanrong and Mingkong, 2014; Laibiao et al., 2015; Haibo, 2016; Di et al., 2017; Qiaren and Xiaohong, 2018; Tao et al., 2018; Dianrong et al., 2020) mentioned the SCV of common peroneal nerve. With the heterogeneity test used, there was considerable heterogeneity between the research (*p* < 0.10, I^2^ = 96%). We combined effect size using a random effects model. and we found that the intervention group outperformed the control group in terms of increasing the SCV of the common peroneal nerve, and this difference was statistically significant [MD = 4.16, 95% CI (1.62, 6.70), *p* = 0.001; Figure 6]. Subgroup analysis showed that there were remarkable differences in intervention time (*p* < 0.05), intervention method (*p* < 0.05), and sample size (*p* < 0.05) among subgroups (Table 3).

##### 3.3.2.4 MCV of common peroneal nerve

**FIGURE 7:**
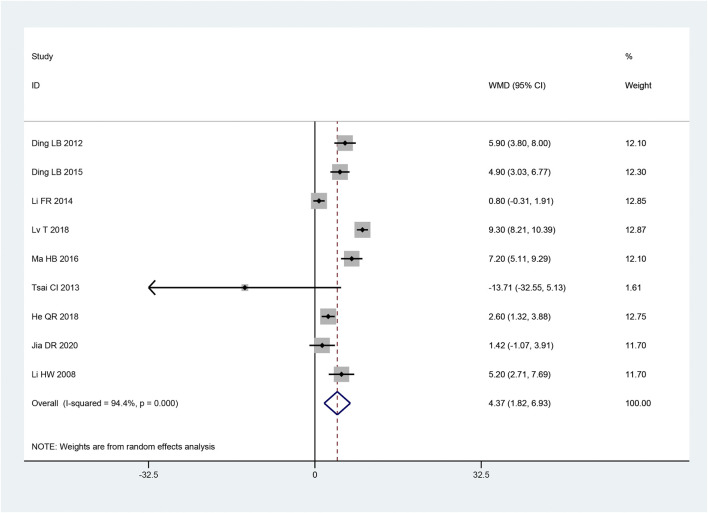
Forest plot of MCV of Common Peroneal Nerve.

In 9 articles (Hongwei et al., 2008; Laibiao et al., 2012; Tsai et al., 2013; Fanrong and Mingkong, 2014; Laibiao et al., 2015; Haibo, 2016; Qiaren and Xiaohong, 2018; Tao et al., 2018; Dianrong et al., 2020), the MCV of the common peroneal nerve was described. According to the heterogeneity test, there was considerable heterogeneity between the papers (*p* < 0.10, I^2^ = 94%). The intervention group significantly improved the MCV of the common peroneal nerve compared to the control group, and it was statistically significant that the findings of this investigation, according to the results of the random effect model used to combine the effect size [MD = 4.37, 95% CI (1.82, 6.93), *p* = 0.0008; Figure 7]. While the intervention time (*p* = 0.056) was not statistically significant, subgroup analysis revealed that there were significant differences in the intervention method (*p* < 0.05) and sample size (*p* < 0.05) in the intervention group (Table 3).

##### 3.3.2.5 SCV of sural nerve

**FIGURE 8:**
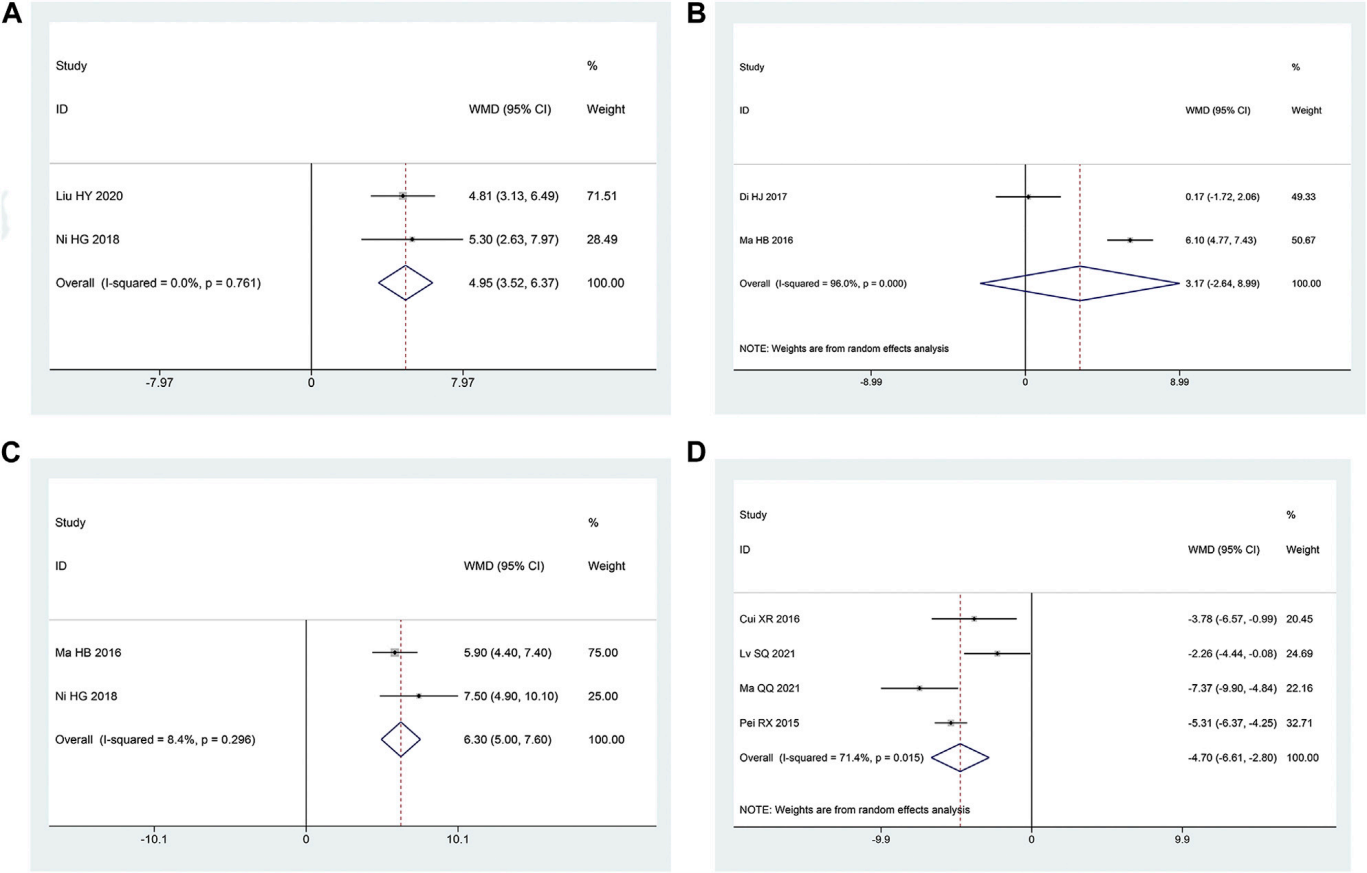
Forest plot **(A)** SCV of Sural Nerve; **(B)** SCV of Tibial Nerve; **(C)** MCV of Tibial Nerve; **(D)** TCM Syndrome Score.

Two articles (Honggang et al., 2018; Haiyan and Yange, 2020) mentioned the SCV of sural nerve. According to the heterogeneity test, there was homogeneity between the studies (*p* = 0.76, I^2^ = 0%). The effect size was combined using the fixed effect model, and the findings demonstrated that the intervention group had a greater benefit in improving the SCV of the sural nerve, with the difference being statistically significant [MD = 4.95, 95% CI (3.52, 6.37), *p* < 0.05; Figure 8A].

##### 3.3.2.6 SCV of tibial nerve

Two articles (Haibo, 2016; Di et al., 2017) reported the SCV of tibial nerve. Following the heterogeneity test, there was significant homogeneity between the papers (*p* < 0.10, I^2^ = 96%). The experimental group had advantages in terms of enhancing tibial nerve SCV, but the results of this experiment were not statistically significant, according to the results of the random effect model that was used to combine the effect size [MD = 3.17, 95% CI (-2.64, 8.99), *p* = 0.28; Figure 8B]. The two studies had a large gap in the intervention time and the intervention method of the intervention group, and the heterogeneity may be related to this.

##### 3.3.2.7 MCV of tibial nerve

Two articles (Haibo, 2016; Honggang et al., 2018) were involved with the MCV of tibial nerve. According to the heterogeneity test, there was homogeneity amongst the studies (*p* = 0.30, I^2^ = 8%). Hence, we integrate the effect sizes by using the fixed effects model. The results revealed that the experimental group had more advantages than the control group in terms of enhancing the MCV of the tibial nerve, and the findings of this experiment were statistically meaningful. [MD = 6.30, 95% CI (5.00, 7.60), *p* < 0.05; Figure 8C].

#### 3.3.3 Clinical effective rates

**FIGURE 9:**
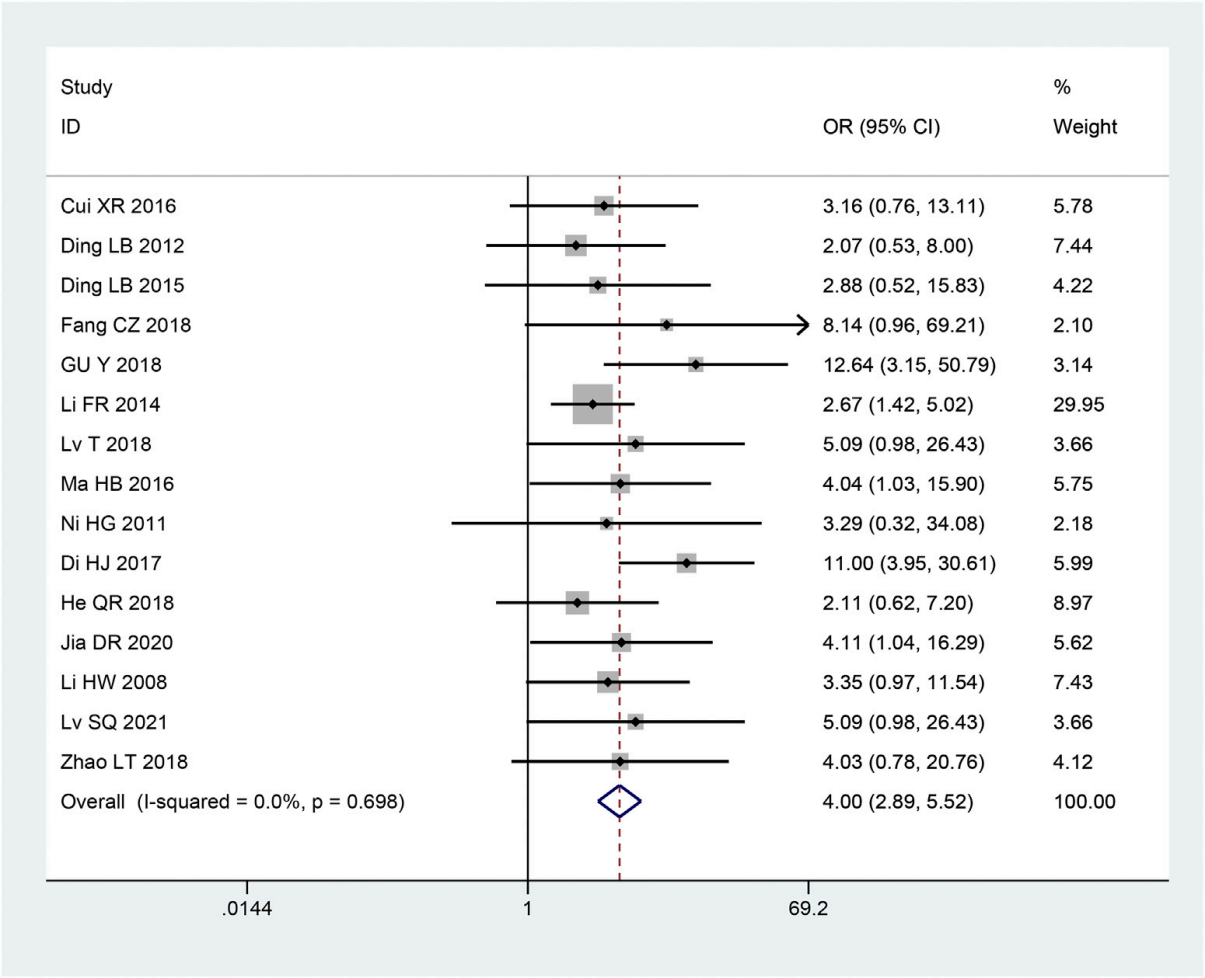
Forest plot of Clinical Effective Rates.

There were 15 articles (Hongwei et al., 2008; Honggang, 2011; Laibiao et al., 2012; Fanrong and Mingkong, 2014; Laibiao et al., 2015; Haibo, 2016; Xiaorui, 2016; Di et al., 2017; Cizhen, 2018; Liting et al., 2018; Qiaren and Xiaohong, 2018; Tao et al., 2018; Ye et al., 2018; Dianrong et al., 2020; Shuquan et al., 2021) involving clinical effective rates. Based on the heterogeneity test, studies were homogeneous (*p* = 0.698, I^2^ = 0%). Thus, to combine the effect sizes, we employ a fixed effects model. According to the findings, the intervention group’s clinical effectiveness rate was higher than that of the control group, and this difference was statistically significant [OR = 4.00, 95% CI (2.89, 5.52), *p* < 0.05; Figure 9].

#### 3.3.4 TCM syndrome score

Four articles (Ruixia et al., 2015; Xiaorui, 2016; Qingqing, 2021; Shuquan et al., 2021) mentioned TCM syndrome scores. The heterogeneity test revealed a significant quantity of heterogeneity among research (*p* = 0.01, I^2^ = 71%). Hence, we integrate the effect sizes by using the random effects model. The findings revealed that the experimental group had a bigger benefit over the control group in terms of improving TCM syndromes and it was statistically significant that the difference [ MD = -4.70, 95% CI (-6.61, -2.80), *p* < 0.05; Figure 8D]. Significant differences between the intervention type subgroups were found in the subgroup analysis. (*p* = 0.009) (Table 3).

### 3.4 Adverse events

Among the 21 papers included, adverse occurrences were described in 5 pieces of literature (Tsai et al., 2013; Laibiao et al., 2015; Ruixia et al., 2015; Xiaorui, 2016; Qingqing, 2021), covering liver function, renal function, blood routine, urine routine, and digestive system. Four of them (Laibiao et al., 2015; Ruixia et al., 2015; Xiaorui, 2016; Qingqing, 2021) stated that the intervention group’s incidence of negative events was lower compared to the western medicine intervention group. One paper (Tsai et al., 2013) reported that the intervention group had more negative occurrences than the placebo group did. The commonest adverse reactions were stomach discomfort, nausea, dry mouth, etc. However, all adverse reactions were not treated specially, and the symptoms gradually relieved or disappeared. Although the above results suggest that CHM in the treatment of PDN is safe because the sample size is small, more large sample clinical studies are needed to prove the conclusion.

### 3.5 Sensitivity analysis

Individual studies were excluded one by one for sensitivity analysis. The findings indicate that after removing the studies, there was no substantial change in the outcomes for any outcome indicators, revealing that the results were stable.

### 3.6 Publication bias

**FIGURE 10:**
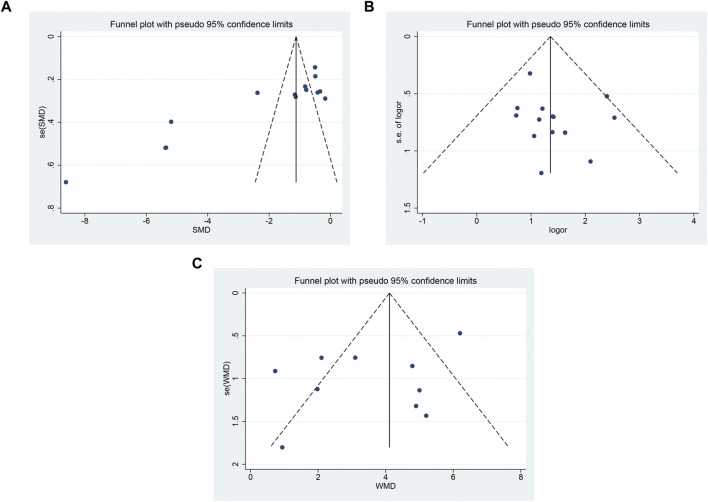
The funnel plot **(A)** pain score; **(B)** clinical effective rate; **(C)** SCV symmetry of median nerve.

The funnel plot showed that there was asymmetry in the pain score (Figure 10A), and the symmetry of the clinical effective rate (Figure 10B) and the symmetry of the median nerve SCV (Figure 10 C) were acceptable. Using Begg’s test and Egger’s test, it showed that there was a substantial publication bias in the pain ratings (*p* = 0.002; *p* < 0.05), but not in the clinical effective rate (*p* = 0.216; *p* = 0.357) or SCV of the median nerve (*p* = 0.721; *p* = 0.157).

## 4 Discussion

### 4.1 Summary of main results

In this study, a total of 492 publications were found, and 21 articles were used for the meta-analysis. The primary findings of this meta-analysis demonstrated that CHM, whether used as an adjuvant therapy or a stand-alone treatment, enhanced nerve conduction velocity and clinical efficacy during the therapy of PDN and reduced pain scores and TCM syndrome scores. To identify the reasons for heterogeneity, we carried out subgroup analyses based on intervention time, intervention methods, sample size, and so on. The findings of the subgroup analysis demonstrated that one of the reasons of heterogeneity in the SCV of the median nerve, SCV of the common peroneal nerve, and pain score was the intervention time. The sample size is one of the heterogeneous sources of SCV of median nerve, SCV of common peroneal nerve, MCV of common peroneal nerve, and MCV of median nerve. The pain rating scale is also one of the heterogeneous sources of pain scores. The study’s results are steady and dependable, according to sensitivity analysis. According to the publication bias test, there is a risk of bias in this study. In addition, we found that adverse events of CHM treatment are less than conventional western medicine treatment, indicating that Chinese herbal treatment of PDN is safe. Therefore, we provide supporting evidence that CHM is effective and safe in treating PDN.

### 4.2 Frequency analysis of Chinese herbal medicine

A total of 72 Chinese medicines were involved in all formulations. Ranked according to the frequency of Chinese herbal medicines, the top 15 flavors of Chinese medicine frequency distribution are shown in Table 4, of which *Astragalus mongholicus* Bunge [Fabaceae; Astragali radix], *Angelica sinensis* (Oliv.) Diels [Apiaceae; Angelicae sinensis radix], and *Cnidium monnieri* (L.) Cusson [Apiaceae; Chuanxiong rhizoma] frequency rank among the top three, whose frequency is more than 10 times. This conclusion agrees with the findings of Zhang Fuzhi et al. (Fuzhi et al., 2020).

**TABLE 4:** Frequency distribution of CHM.

Chinese name	Accepted name	Family	Frequency
Huangqi	*Astragalus mongholicus* Bunge	Fabaceae	13
Danggui	*Angelica sinensis* (Oliv.) Diels	Apiaceae	12
Chuanxiong	*Cnidium monnieri* (L.) Cusson	Apiaceae	11
Chishao	*Paeonia lactiflora* Pall.	Paeoniaceae	10
Gancao	*Glycyrrhiza glabra* L.	Fabaceae	10
Niuxi	*Achyranthes bidentata* Blume	Amaranthaceae	10
Jixueteng	*Spatholobus suberectus* Dunn	Fabaceae	9
Guizhi	*Neolitsea cassia* (L.) Kosterm.	Lauraceae	8
Baishao	*Paeonia lactiflora* Pall.	Paeoniaceae	7
Danshen	*Salvia miltiorrhiza* Bunge	Lamiaceae	7
Dilong	*Pheretima vulgaris* Chen	Earthworms	7
Taoren	*Prunus persica* (L.) Batsch	Rosaceae	7
Yanhusuo	*Corydalis yanhusuo* (Y.H.Chou & Chun C.Hsu) W.T.Wang ex Z.Y.Su & C.Y.Wu	Papaveraceae	7
Fuling	*Wolfiporia cocos* (F.A. Wolf) Ryvarden & Gilb.	Polyporus	6
Honghua	*Carthamus tinctorius* L.	Asteraceae	6

Chemical constituents are the key material basis for revealing the efficacy of CHM (Chuan, 2017). Astragaloside IV, which is the main chemical component of *Astragalus*, can inhibit Schwann cell apoptosis, regulate nerve growth factor gene expression, increase NA + -K + -ATPase, inhibit erythrocyte aldose reductase activity, thereby inhibiting nerve injury, enhancing the speed of motor nerve conduction and minimizing the buildup of nerve and erythrocyte advanced glycation end products (Yuanyuan et al., 2021). Xie et al. have shown that quercetin, a common flavonoid compound in astragalus, can regulate the expression of intestinal flora and reactive oxygen species in diabetic peripheral neuropathy rats to improve peripheral nerve injury (Xie et al., 2020). Isoeugenol, one of the main components of Angelica Sinensis volatile oil, plays an important role in reducing oxidative stress markers and glutathione levels in the sciatic nerve area (Prasad and Muralidhara, 2013). Additionally capable of removing oxygen free radicals and minimizing peroxide damage are ferulic acid and angelica polysaccharide (Gong et al., 2016). One of the key ingredients of Chuanxiong Rhizoma is ligustrazine, which has the ability to up-regulate the expression of heme oxygenase 1/carbon monoxide and superoxide dismutase and down-regulate the expression of tumor necrosis factor-, nitric oxide synthase/nitric oxide, and malondialdehyde. It is proved that ligustrazine can enhance the body’s antioxidant and anti-inflammatory effects and play a protective role in diabetic pain neuropathy (Fangqin et al., 2021).

Wnt protein is an important signalling molecule. The wnt signalling pathway mediates a variety of biological processes in the body, such as embryogenesis, organogenesis and tumour formation (Sharma et al., 2022). Among them, the wnt signalling pathway is involved in the development of T2DM and its complications by directly influencing the differentiation and proliferation of pancreatic β-cells as well as the secretion and action of insulin (Nie et al., 2021). This provides a direction to explore the mechanism of action of CHM in the treatment of diabetes and its complications. It (Zou et al., 2016; Wang et al., 2020; Zhang et al., 2020; Wu et al., 2022) was found that astragaloside, angelica polysaccharide and chuanxiongzine could all activate or inhibit the wnt signalling pathway in tissues or cells to regulate glucose and lipid metabolism, tissue or organ repair in T2DM patients.

In addition, the traditional Chinese medicine involved in this study is not only effective in the treatment of PDN, but also has a good effect on type 2 diabetes and other complications (such as diabetic heart disease, diabetic retinopathy). Studies have found that ligustrazine can delay the development of DR by inhibiting oxidative stress and retinal ganglion cell apoptosis, down-regulating AGEs content and other ways (Wei, 2021). *Astragalus* polysaccharide alleviated mitochondrial damage and apoptosis induced by metabolic memory by regulating the miR-182/Bcl-2 axis (Nie et al., 2019). Astragaloside IV improves endothelial dysfunction in thoracic aortas from diabetic rats by reducing oxidative stress and calpain-1 (Gao et al., 2021).

### 4.3 Advantages and limitations of research

Although there are more and more clinical studies on the treatment of PDN with CHM, there is no systematic evaluation and meta-analysis in this direction. This study is the first study to systematically evaluate CHM for PDN, filling the evidence-based gap in CHM for PDN. The advantages of this meta-analysis mainly include a clear research topic, its selects high-quality RCTs that meet the inclusion criteria and conducted a statistical analysis of this study in strict accordance with the systematic review method. At the same time, we are more cautious about the explanation of the results. We conducted a subgroup analysis to find out the cause of heterogeneity, and we also conducted a sensitivity analysis and publication bias test. This study found that CHM can be recommended for the treatment of PDN, which offers fresh perspectives and ideas for researching PDN.

However, there are a few shortcomings in this work that deserve discussion. First of all, the majority of the included researchers did not use the allocation concealment and blind method, which could cause bias in both selection and implementation. Second, there was significant clinical heterogeneity in the 21 studies with differences in composition, dose, and dosage form of CHM, as well as differences in interventions (type of WM) and duration of intervention in the control group. This would result in a high level of heterogeneity within subgroups when subgroup analyses are performed. However, because of the limited number of studies, we cannot perform relevant subgroup analysis, which in turn affects the accuracy of the results. Third, the duration of the study’s intervention ranged from 4 to 12 weeks, and we could not assess the long-term safety of CHM treatment. Finally, since the randomized controlled trials included in this study are all from China, our study may not be extended to the world. Therefore, a large sample of multi-center studies is needed in the future.

## 5 Conclusion

In short, CHM, whether a single treatment or adjuvant therapy, can improve nerve conduction velocity in patients with PDN, reduce pain score and TCM syndrome score, and improve clinical efficiency. These results can guide clinical practice. In addition, CHM is well tolerated and safe in patients with PDN, with a low incidence of adverse events. However, given the study’s heterogeneity and small sample size, bigger multi-center, high-quality RCTs will be required in the future to evaluate the advantages and safety of CHM in the treatment of PDN.

## Data Availability

The original contributions presented in the study are included in the article/Supplementary Material, further inquiries can be directed to the corresponding author.
